# Enhanced therapeutic efficacy of LHRHa-targeted brucea javanica oil liposomes for ovarian cancer

**DOI:** 10.1186/s12885-016-2870-4

**Published:** 2016-10-29

**Authors:** Hongxia Ye, Xiaojuan Liu, Jiangchuan Sun, Shenyin Zhu, Yi Zhu, Shufang Chang

**Affiliations:** 1 Department of Obstetrics and Gynecology, Second Affiliated Hospital of Chongqing Medical University, No. 74 Linjiang Road, Yuzhong district, Chongqing, 400010 China; 2 Department of Pharmacy, First Affiliated Hospital of Chongqing Medical University, No. 1 Youyi Road, Yuzhong district, Chongqing, 400010 China

**Keywords:** Liposomes, Targeted, Luteinizing hormone releasing hormone, Brucea javanica oil, Ovarian cancer

## Abstract

**Background:**

Although brucea javanica oil liposomes (BJOLs) have been used clinically to treat ovarian cancer, its clinical efficacy is often limited by systemic side effects due to non-specific distribution. Luteinizing hormone releasing hormone receptor (LHRHR) is overexpressed in most ovarian cancers but negligibly expressed in most of the other visceral organs. In this study, we aimed to develop a novel LHRHa targeted and BJO-loaded liposomes (LHRHa-BJOLs), and investigate its characteristics, targeting ability and anti-ovarian cancer efficiency both in vitro and in vivo.

**Methods:**

The LHRHa-BJOLs were prepared by film-dispersion and biotin-streptavidin linkage methods, and characterized in terms of its morphology, particle size, zeta potential, ligand conjugation, encapsulation efficiency and stability. The targeting nature and antitumor effects of the liposomes were evaluated in vitro using cultured human ovarian cancer A2780/DDP cells, and in vivo using ovarian cancer-bearing nude mice.

**Results:**

The LHRHa-BJOLs were successfully synthesized, with a uniformly spherical shape, appropriate particle size and zeta potential, as well as a high encapsulation efficiency. Compared to non-targeted liposomes and BJO emulsion, the LHRHa-BJOLs could significantly increase specific intracellular uptaking rate, enhance cell inhibitory effect and induce cell apoptosis in A2780/DDP cells in vitro. Meanwhile, LHRHa-BJOLs also had a significantly stronger activity of targeting tumor tissue, inhibiting tumor growth, inducing tumor apoptosis and prolonging survival time in ovarian cancer-bearing mice in vivo.

**Conclusions:**

Our experiment suggests that LHRHa-BJOLs may be a useful targeted drug for the treatment of ovarian cancer.

## Background

Ovarian cancer is the leading cause of death from gynecologic malignancy [1–3], and is a silent killer with stable incidence and poor prognosis due to the difficulties of early diagnosis [4]. The treatment of ovarian cancer usually involves debulking surgery and chemotherapy [5, 6]. Seed oil of Brucea javanica (BJO), a traditional herbal medicine, is extracted from the seeds of Brucea javanica [7], and has been used clinically to treat various tumors, including ovarian cancer [7–9]. The mechanisms of antitumor activity of BJO include inducing tumor apoptosis and reversing multidrug resistance [10, 11]. However, its clinical use is limited due to non-specific distribution, low therapeutic index and systemic side effects [8].

Considerable research has been carried out to overcome these defects, including develop improved dosage and drug delivery systems. Liposomes have been developed as an effective drug carrier due to their ability to deliver encapsulated drugs to specific target sites, provide sustained drug release and protect encapsulated agents [12–14]. Furthermore, many targeting ligand-conjugated liposomes have been tested in recent years, including natural or synthetic ligands. Luteinizing hormone releasing hormone receptor (LHRHR) is overexpressed in approximately 70–80 % of ovarian cancer cells, however, its expression in most of the other visceral organs is negligible [15–17]. Since natural LHRH is unstable in vivo, LHRH analogue (LHRHa) with improved bioactivity has been synthesized as an ovarian cancer seeking agent to target LHRHR [18]. Previous animal studies have shown that an LHRHa targeted drug delivery system exhibits no pituitary toxicity, insignificant influence on the luteinizing hormone concentration, and negligible effect on the reproductive functions [17]. In addition, LHRHa is a stable protein with well-defined reaction sites for conjugation, and has been shown to undergo receptor-mediated endocytosis, and transporting the ligand-receptor complex into the cells [18, 19]. More recently, we have successfully synthesized an LHRHa conjugated and paclitaxel loaded microbubbles for ultrasound mediated chemotherapy that induced ovarian cancer apoptosis in vitro and in vivo [20–23].

In this study, we coupled the LHRHa ligands with BJO-loaded liposomes (LHRHa-BJOLs) to target human ovarian cancer A2780/DDP cells that express the LHRHR. The BJO loading rate in liposomes was detected by an ultraviolet spectrophotometry, and the LHRHa in BJO loaded liposomes (BJOLs) was detected by immune colloidal gold technique and observed by transmission electron microscopy. The targeted uptaking of LHRHa-BJOLs to cancer cells was observed by fluorescence microscope and analyzed by flow cytometry. The anticancer effects of LHRHa-BJOLs were then tested in vitro and in vivo. Our experiment verifies the hypothesis that LHRHa-BJOLs will enhance the efficacy of BJO therapy. To the authors’ knowledge, no similar study has been reported elsewhere.

## Methods

### Cell lines and culture

Human ovarian cancer A2780/DDP cells (LHRHR positive [20]) were kindly provided by Professor Zehua Wang from Wuhan Union Hospital (Wuhan, China). SKOV3 cells (LHRHR negative [22]) were obtained from School of Life and Health Sciences, Chongqing Medical University (Chongqing, China). The cells were grown in RPMI-1640 medium (HyClone, Utah, USA) at 37 °C under a humidified atmosphere of 5 % CO_2_, and supplemented with 10 % fetal bovine serum (Tianhang Biotechnology Co., Ltd., China) and 1 % penicillin-streptomycin.

### Animal model preparation

Female BALB/c-nu/nu nude mice (4–5 weeks old; body weight, 18–20 g) were purchased from the Animal Centre of Chongqing Medical University and housed in laminar flow rooms under constant temperature (22 ± 2 °C), humidity and specific pathogen-free conditions. Tumors were established by subcutaneous injection of A2780/DDP cells. A2780/DDP cells were suspended in serum-free RPMI-1640 medium to reach a cell density of 4 × 10^7^/ml and injected subcutaneously into the left flanks of the mice at a dose of 0.2 ml, tumors were inspected by observation and palpation. All animal experiments were approved ethically and scientifically by the Chongqing Medical University in accordance with the Practice Guidelines for Laboratory Animals of China.

### Preparation of LHRHa-BJOLs

LHRHa-BJOLs were prepared by the film-dispersion and biotin-streptavidin linkage methods. Briefly, the mixtures of Soybean phosphatidylcholine (Lipoid Co., Ltd., Germany), Cholesterol (Sigma Aldrich Co., Ltd., St. Louis, USA), 1,2-distearoyl-sn-glycero-3-phosphoethanol-amine-N-[biotinyl (polyethyleneglycol) (2000)] (DSPE-PEG2000-Biotin, Avanti Polar Lipids Co., Ltd., Alabaster, USA) and BJO (Yaoda Pharmaceutical Co., Ltd., Shenyang, China) (194:50:6:75, w/w) were fully dissolved in sufficient chloroform and methanol (5:1, v/v) in a round bottom flask. The organic solvent was removed by rotary evaporation (37 °C, 100 r/min, 5Kpa, 30 to 40 min) and the resulting film was dried by storing in a vacuum overnight. After hydrating the film in 3 ml phosphate buffered saline (PBS, PH 7.4, 290 mosm, Zhongshanjinqiao Biotechnology Co., Ltd., Beijing, China), the solution was sonicated for 8 min at 4 °C in an ultrasonic water bath. The suspensions were then filtered by 0.45 μm and 0.22 μm microfiltration membrane in sequence, and the BJOLs were obtained. Streptavidin (SA, SA: DSPE-PEG_2000_-Biotin = 1:8, molar ratio) was added to the BJOLs solution and incubated at 4 °C with gentle stirring for 30 min. An excessive amount of the biotinylated LHRHa (Beijing Zhongkeyaguang Biotechnology Co., Ltd., Beijing, China) was then added into the solution and gentle stirred at 4 °C for another 30 min to form the LHRHa-BJOLs. Finally, Sephadex G-50 column (Pharmacia Biotech Inc., New Jersey, USA) was used to separate LHRHa-BJOLs from the free streptavidin and LHRHa peptide. Coumarin-6 loaded liposomes (C6Ls) and LHRHa-modified C6Ls (LHRHa-C6Ls), being used as fluorescently labelled probes, were prepared as described above by substituting the BJO with coumarin-6 (Sigma Aldrich Co., Ltd., St. Louis, USA).

### Characterization of LHRHa-BJOLs

The particle size and zeta potential were determined using dynamic light scattering with the Zetasizer Nano ZS90 (Malvern Instrument Ltd., Worcestershire, UK), samples were diluted appropriately with double distilled water for the measurements. The content of BJO in liposomes was measured by ultraviolet spectrophotometry. LHRHa-BJOLs suspensions (1 ml) were eluted by PBS in Sephadex G-50 column, and then the opalescence part of the eluate was collected. The amount of BJO in the suspensions before and after passing over the Sephadex G-50 column (W_total_ and W_free_) were measured. The encapsulation efficiency (EE) of BJO in liposomes was calculated with the formula: EE = (W_total_ - W_free_)/W_total_ × 100 %.

### Stability of LHRHa-BJOLs

LHRHa-BJOLs were stored at 4 and 25 °C in the dark for 3, 6, 9, 12, 15 and 30 days, respectively. The percolation rate (PR) of BJO in different storage conditions was calculated by the following equation: PR = (EE_0_ - EE)/EE_0_ × 100 %, where the EE_0_ and EE were the encapsulation efficiency of BJO before and after storage, respectively.

### Measurement of LHRHa on the surface of LHRHa-BJOLs

The LHRHa on the BJOLs’ surface was detected by immune colloidal gold technique and observed by transmission electron microscopy (TEM, H-7500, JEOL, Japan). Briefly, the rabbit anti-human LHRH monoclonal antibody (Chemicon International, Inc., USA) was mixed with the LHRHa-BJOLs and BJOLs, respectively, followed by an incubation at 4 °C for overnight. After passed over the Sephadex G-50 column, the suspensions were incubated with goat anti-rabbit IgG labeled with 15 nm immunogold nanoparticles (Bioss Biotechnology Co., Ltd., Beijing, China) at room temperature for 90 min, and then washed with PBS on a Sephadex G-50 column and spread over a carbon-coated copper grid, negatively stained with 1 % phosphotungstic acid and allowed to dry. The morphology of the LHRHa-BJOLs was then observed using TEM.

### In vitro study of the targeted binding affinity

The intracellular uptake of the LHRHa-Liposomes (LHRHa-Lip) was observed by laser scanning confocal microscope (LSCM, Leica, Heidelberg, Germany) and coumarin-6 was used as the fluorescent probe. Briefly, A2780/DDP and SKOV3 cells were seeded in 6-well plate in triplicate at a density of 4 × 10^5^/well and incubated for 24 h, respectively. The medium was then changed with fresh culture medium containing either LHRHa-C6Ls or C6Ls at a coumarin-6 concentration of 2 μg/ml. After incubated for 1.5 h at 37 °C and washed three times with pre-cooling PBS to remove unbound liposomes, the cells were fixed in 4 % paraformaldehyde at 4 °C for 15 min and washed with PBS. After that, the cells were stained with DAPI-containing reagent for 5 min and washed with PBS to remove the free DAPI. The uptaking of LHRHa-C6Ls or C6Ls in the cancer cells were observed by LSCM.

The intracellular uptake of LHRHa-Lip was also quantitatively analyzed by flow cytometry (FCM, FACS, BD Biosciences, San Jose, CA, USA). Briefly, A2780/DDP and SKOV3 cells were seeded in 6-well plate in triplicate at a density of 4 × 10^5^/well and incubated. When 80 % confluence was reached, the cells were washed with PBS, and the LHRHa-C6Ls or C6Ls (containing 2ug/ml coumarin-6) was added, respectively. After 1.5 h incubation, the cells were washed three times with PBS, harvested with trypsin, centrifuged, and resuspended at a concentration of 1 × 10^6^/ml in culture medium. The mean fluorescent intensity (MFI) of the cells were analyzed by FCM.

### In vitro evaluation of antitumor efficiency

A2780/DDP cells were seeded in 6-well plate (5 × 10^5^ cells/well) in triplicate and incubated for 24 h to allow cell adhesion. After that, the cells were equally divided into the following four treatment groups: PBS, BJOE, BJOLs and LHRHa-BJOLs groups. For BJOE, BJOLs and LHRHa-BJOLs groups, BJO was administered at a dose of 0.4 mg/ml.

### Cell viability assay

After different treatments, the cells were incubated for a further 24, 48 and 72 h and washed in PBS for three times. The number of viable cells in each treatment group relative to PBS group was evaluated using a 3-(4, 5-dimethylthiazol-2-yl)-2, 5-diphenyltetrazolium bromide (MTT) assay (Sigma Aldrich Co., Ltd., St. Louis, USA). Briefly, at these designated time points, 20 μl MTT (5.0 mg/ml) was added and the cells were incubated for another 4 h. Then, the medium was removed, 150 μl dimethylsulfoxide was added and mixed on a vortex mixer for 10 min. Absorbance (A) at 490 nm was recorded using the microplate reader (BIO-RADModle 550, Bio-Rad Ltd., USA). The cell inhibitory rate (IR) was calculated by the following equation: IR (%) = [1 - (A_treated_/A_control_)] × 100 %.

### Apoptosis assay

Twenty-four hours after different treatments, the cell apoptosis was assessed using an Annexin V-FITC/PI apoptosis detection kit (Beyotime Biotechnology Co., Ltd., Jiangsu, China) as described by the manufacturer’s instructions. Briefly, A2780/DDP cells treated with various treatments were harvested with trypsin, washed twice with PBS, resuspended in Annexin-V binding buffer and incubated in the dark for 15 min with 5 μl AnnexinV-FITC and 10 μl PI. The apoptosis was evaluated by FCM, and the apoptosis rate of each group was calculated using Cell Quest software.

### JC-1 mitochondrial membrane potential (MMP) assay

Twenty-four hours after different treatments, the cell MMP (Δψm) was assessed using a JC-1 MMP assay kit (Beyotime Biotechnology Co., Ltd., Jiangsu, China) according to the manufacturer's instructions. Briefly, A2780/DDP cells treated with various treatments were collected and washed with PBS, and incubated with 0.5 ml JC-1 dye in the dark for 20 min at 37 °C. The cells were then washed twice with JC-1 buffer and then analyzed by FCM.

### Detection of apoptosis by Hoechst 33258 fluorescent staining

Twenty-four hours after different treatments, A2780/DDP cells were washed with PBS, fixed with paraformaldehyde (Sigma, St. Louis, MO, USA) at room temperature for 15 min and washed with PBS, then stained in Hoechst 33258 dye (4 g/ml, Beyotime Institute of Biotechnology, China) in dark for 5 min at 37 °C. After been washed with PBS for three times, the cells were observed under fluorescence microscopy. Apoptotic cells were defined with the changes of nuclear morphology. Normal nuclei showed diffusely and homogeneously low-intensity fluorescent, apoptotic nuclei were hyperchromatic and compact at condensed or granular state.

### Bcl-2, Bax and caspase-3 activity

In order to further understand the apoptosis-inducing effect of LHRHa-BJOLs in ovarian tumor cells, apoptosis-related proteins, including bcl-2, bax and caspase 3, were evaluated in A2780/DDP cells at 24 h after different treatments by western blot analysis. The cells in each group were lyzed in a lysis buffer (50 mM Tris–Cl, pH 7.4, 1 mM EDTA, 150 mM NaCl, 1 % NP40, 0.25 % Na-deoxycholate, and 1 μg/ml of aprotinin, leupeptin and pepstatin). Equal amounts of protein (30 μg/sample) were separated electrophoretically by 15 % SDS-PAGE and blotted onto a polyvinylidene difluoride membrane, which was then blocked with PBS containing 5 % non-fat dried milk for at least 1 h. The blots were incubated at 4 °C overnight with a primary antibody against bcl-2 (Rabbit anti-human bcl-2 antibody, CST, Inc., USA), bax (Rabbit anti-human bax antibody, EPI, Inc., USA) and caspase 3 (Rabbit anti-human caspase-3, Santa, Inc., USA) respectively and followed by incubation with a horseradish peroxidase-conjugated secondary antibody for 30 min in a blocking buffer at room temperature. After further washing, the blots were revealed by enhanced chemiluminescence (Pierce ECL detection kit) and exposed to X-ray film (Eastman-Kodak, Rochester, NY, USA). Equal loading was confirmed by β-actin detection. Band optical density (OD) was analyzed using a Labworks 4.6 UVP-image capture and analysis software package. The analysis results were expressed in the format of mean ± standard deviation (SD) as the ratio percentage of the protein of interest OD versus the β-actin OD.

### In vivo evaluation of antitumor efficiency

The anticancer effect of LHRHa-Lip was demonstrated in an ovarian cancer xenografts model which were established as described above. Thirty-two ovarian cancer-bearing mice were randomly divided into the following four treatment groups (seven mice per group): PBS (control), BJOE, BJOLs and LHRHa-BJOLs groups. For each treatment groups, an equivalent BJO dose of 180 mg/kg was injected into the mouse caudal veins on days 15, 18, 21, 24 and 27 after tumor inoculation, respectively. Tumor diameter was measured by a dial caliper once a week until the 42th day after tumor inoculation. The subcutaneous tumor volume was estimated by the following formula: V (mm^3^) = (length × width^2^)/2 [24]. Three mice in each group were sacrificed 24 h after the last treatment and the tumor tissues were harvested for further protein analysis. The rest of the mice (five in each group) were monitored daily for the signs of the reduced physical activity and the progression of the tumor. The survive time of each mouse was recorded.

### Detection of Bcl-2, Bax and caspase-3 expression after in vivo treatment

The bcl-2, bax and caspase 3 protein expression of the tumor were characterized by western blot analysis. Briefly, samples in each treatment group were homogenized and centrifuged at 12000 g for 30 min. The supernatant was collected and the protein concentration of the lysate was determined by a Bradford protein assay (Bio-Rad, Hercules, CA, USA). For western blot analysis, equal amounts of protein were loaded for SDS-PAGE. The protein was then transferred to a nitrocellulose membrane, blocked with PBST (PBS with 0.1 % Tween-20) solution containing 5 % non-fat milk, and incubated overnight at 4 °C with a primary antibody against bcl-2, bax and caspase 3 (polyclonal, 1:1000, Abcam, UK),respectively. The antibody was detected by a horseradish peroxidase-conjugated secondary antibody (1:5000) after 1-hour incubation and developed with an ECL detection kit. Equal loading was confirmed by β-actin detection. Band optical density (OD) was analyzed using a Labworks 4.6 UVP-image capture and analysis software package. The analysis results were expressed in the format of mean ± standard deviation (SD) as the ratio percentage of the protein of interest OD versus the β-actin OD.

### Statistical analysis

Data were analyzed using the Statistical Package for Social Sciences (SPSS) software for Windows, version 22.0 (SPSS Inc., Chicago, USA). One-way analysis of variance (ANOVA) for multiple-group analysis, unpaired student’s *t*-test was used for between two-group comparison, and log-rank test was used for the data of lifetime comparison. The data were expressed as mean value ± standard deviation $\left(\overline{x}\pm s\right)$, a *p* value of less than 0.05 was considered statistically significant.

## Results

### Characterization of the LHRHa-BJOLs

**Fig. 1:**
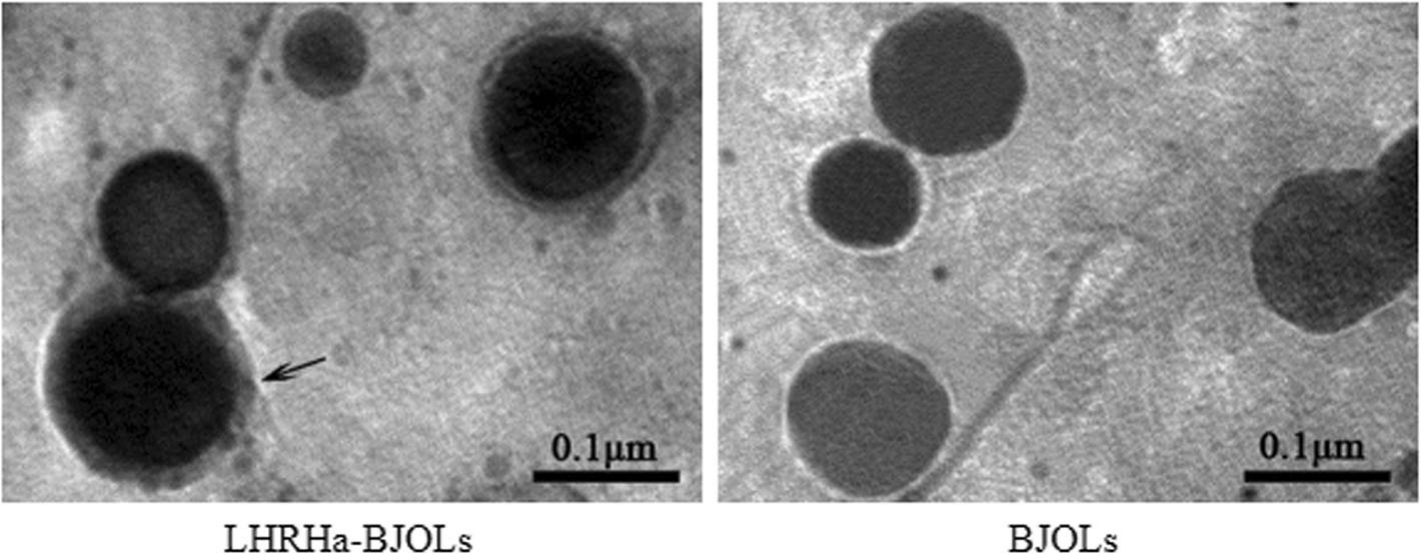
Transmission electron microscopy image of BJO-loaded liposomes. There were obvious black spots (immunogold nanoparticles labeled LHRHa, *black arrow*) on the surface of the LHRHa modified BJO-loaded liposomes, while no black spots on the surface of the BJO-loaded liposomes without LHRHa modification

TEM image showed that the LHRHa-BJOLs were generally spherical in shape, and there were obvious black spots (immunogold nanoparticles labeled LHRHa) on the surface of LHRHa-BJOLs, while no black spots on the surface of BJOLs (Fig. 1), indicating that LHRHa peptide was indeed conjugated on the surface of the LHRHa-BJOLs [25]. The mean size of LHRHa-BJOLs was (155.1 ± 14.5) nm, and the polydispersity index (PDI) was 0.227, indicating a narrow size distribution. The mean zeta potential of LHRHa-BJOLs was - (24.1 ± 0.54) mV. The encapsulation efficiencies (EE) of LHRHa-BJOLs and BJOLs were (92.2 ± 1.59) % and (93.6 ± 1.04) %, respectively. No significant difference in encapsulation efficiencies was observed between LHRHa-BJOLs and BJOLs, indicating that the modification of the liposomes with the LHRHa peptide had no significant influence on the encapsulation efficiencies of the liposomes. After storing in 4 °C in the dark for 3, 6, 9, 12, 15 and 30 days, the percolation rates of LHRHa-BJOLs were 0.12, 0.18, 0.23, 0.67, 1.14 and 1.26 %, respectively. In comparison, when the LHRHa-BJOLs were storied in 25 °C for the same time, the percolation rates were 4.36, 9.52 12.84 15.67 19.25 and 20.08 %, respectively. This indicates that LHRHa-BJOLs were more stable when stored in 4 than 25 °C and it could be stored at 4 °C more than 30 days.

### Intracellular uptake of LHRHa-Lip in vitro

**Fig. 2:**
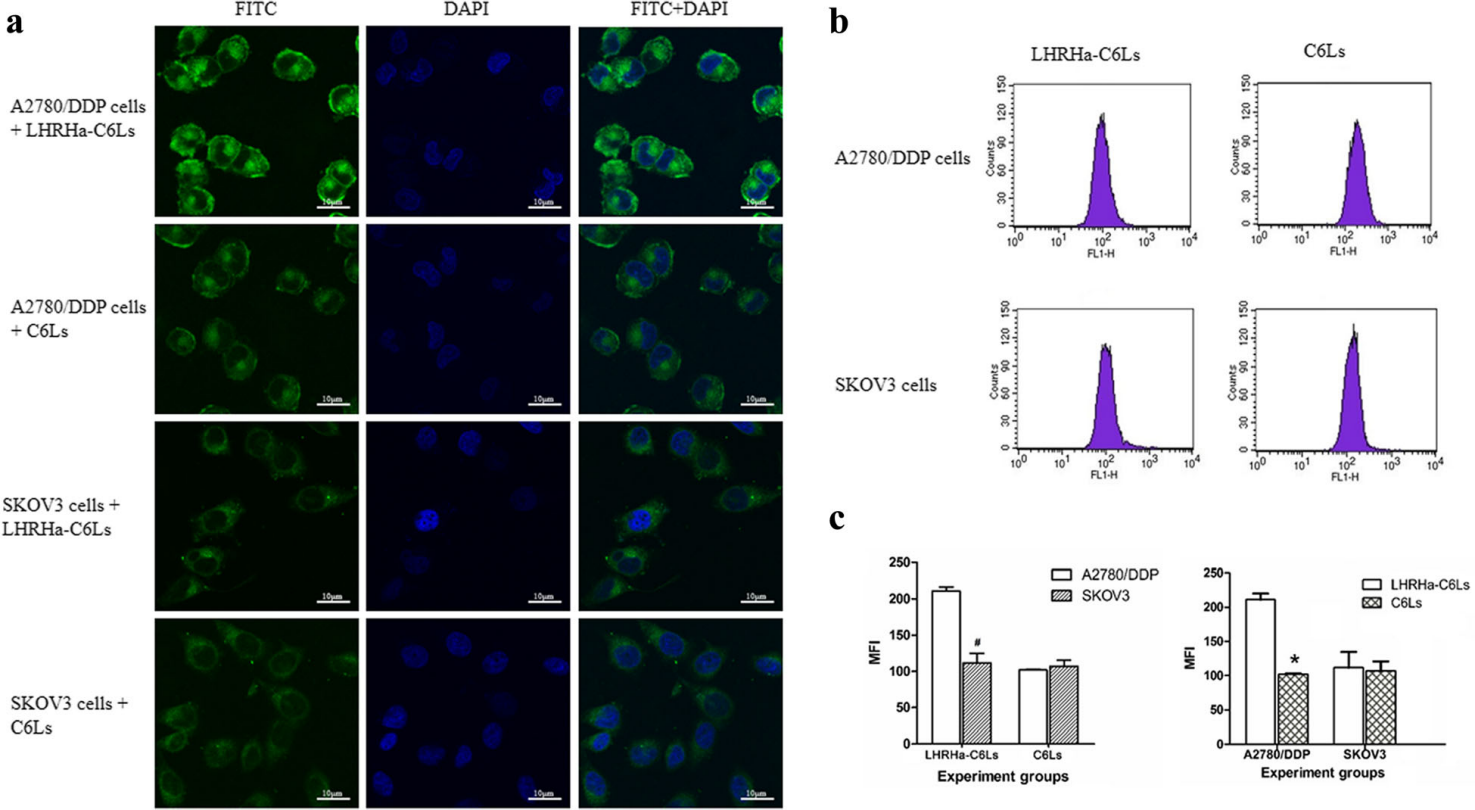
Intracellular uptake of LHRHa-liposomes in vitro. **a** Fluorescence microscopic images acquired for A2780/DDP and SKOV3 cells treated with LHRHa-C6Ls and C6Ls, respectively. A2780/DDP cells treated with LHRHa-C6Ls exhibited higher fluorescence emission than it treated with C6Ls. In comparison, SKOV3 cells treated with LHRHa-C6Ls or C6Ls exhibited the same fluorescence emission. **b** and **c**, Mean fluorescence intensity (MFI) of A2780/DDP and SKOV3 cells by flow cytometry analysis. A2780/DDP cells plus LHRHa-C6Ls group had the highest liposomes uptaking efficiency when compared with other groups (*p* < 0.05). Compared with LHRHa-C6Ls group, *p < 0.05; compared with A2780/DDP cells, #*p* < 0.05

Figure 2a shows the fluorescence microscopic images acquired for A2780/DDP and SKOV3 cells treated with LHRHa-C6Ls and C6Ls, respectively. A2780/DDP cells treated with LHRHa-C6Ls exhibited higher fluorescence emission than it treated with C6Ls, indicating that LHRHa-C6Ls yields the higher liposome uptaking in LHRHR positive A2780/DDP cells. In comparison, SKOV3 cells treated with LHRHa-C6Ls or C6Ls exhibited the same fluorescence emission, indicating that LHRHa-C6Ls doesn’t yield the higher liposome uptaking in LHRHR negative SKOV3 cells. Quantitative analysis of the fluorescence emission intensities by FCM analysis for these treatment groups also yielded consistent conclusions. According to Fig. 2b and c, the fluorescence intensity for A2780/DDP cells treated with LHRHa-C6Ls or C6Ls were (211.09 ± 8.96), and (101.93 ± 1.50), respectively. Whereas those for SKOV3 cells were (111.76 ± 23.22) and (107.04 ± 13.83), respectively. Compared with other treatment groups, A2780/DDP cells treated with LHRHa-C6Ls had the highest liposomes uptaking efficiency and the differences were statistically significant (*p* < 0.05). These experimental results indicate that the increased delivery of LHRHa-C6Ls to A2780/DDP cells is LHRHa mediated.

### Cell viability after LHRHa-BJOLs treatment in vitro

**Fig. 3:**
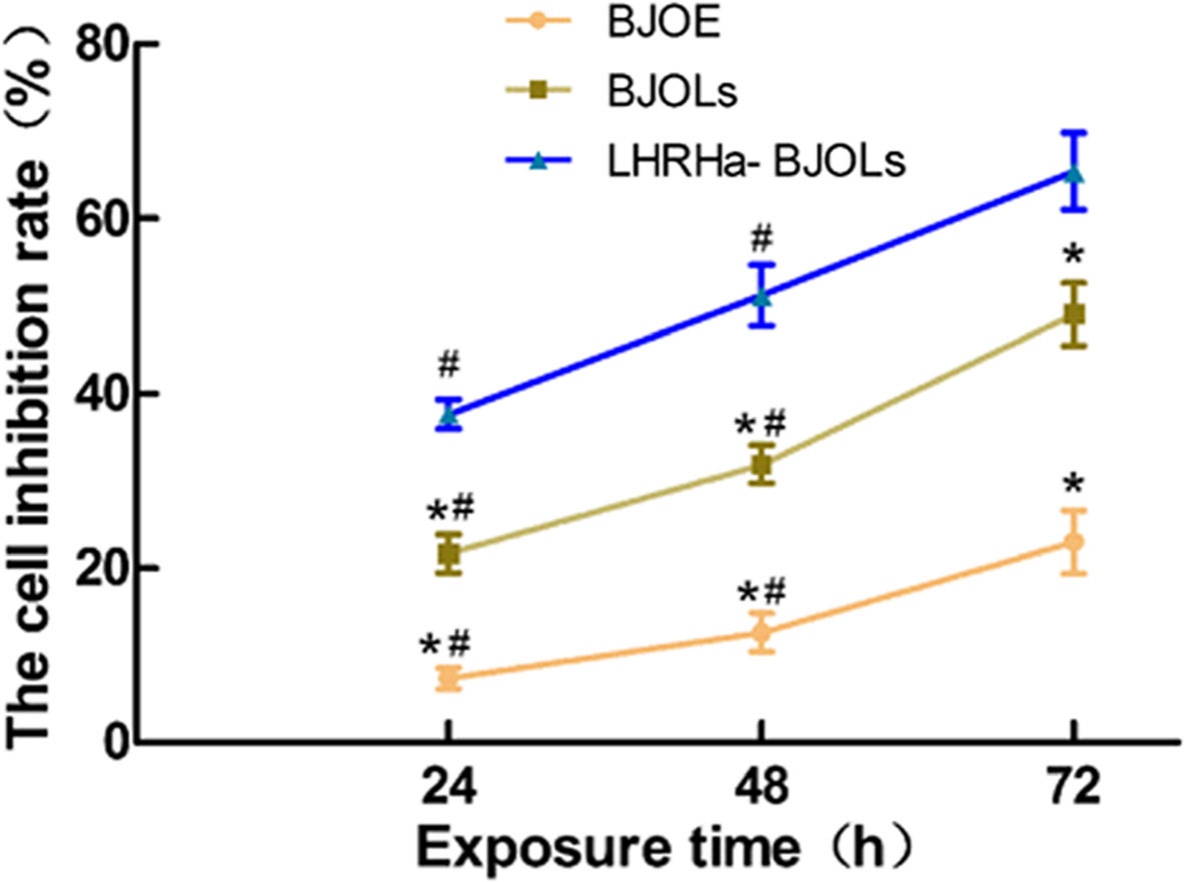
Growth inhibitory effects of different treatments on the proliferation of A2780/DDP cells determined by MTT. The proliferation inhibitory rate of LHRHa-BJOLs group is significantly higher than those of other group (*p* < 0.05). Furthermore, BJOE, BJOLs and LHRHa-BJOLs were found to inhibit A2780/DDP cells in a time-dependent manner. Compared with LHRHa-BJOLs group, *p < 0.05; compared with the same group at 72 h after treatment, #*p* < 0.05

The cytotoxicity profiles of different treatment options (BJOE, BJOLs and LHRHa-BJOLs) was evaluated by a MTT assay and compared with the control treatment (PBS). Figure 3 compares the cell proliferation inhibitory rates at 24, 48 and 72 h after different treatments. According to the figure, the cell proliferation inhibitory rates were less than 30 % for BJOE group. In comparison, BJOLs yielded the cell proliferation inhibitory rates of (21.63 ± 2.19) %, (31.86 ± 2.17) % and (49.12 ± 3.61) %, while LHRHa-BJOLs yielded the cell proliferation inhibitory rates of (37.66 ± 1.73) %, (51.26 ± 3.46) % and (65.45 ± 4.42) % at 24, 48 and 72 h after treatment, respectively. Obviously, LHRHa-BJOLs group resulted in significantly stronger inhibitory effects on the proliferation of A2780/DDP cells. Furthermore, BJOE, BJOLs and LHRHa-BJOLs were found to inhibit A2780/DDP cells in a time-dependent manner.

### Inducing apoptosis in a flow cytometer assay, Hoechst 33258 fluorescent staining and western blotting analysis

**Fig. 4:**
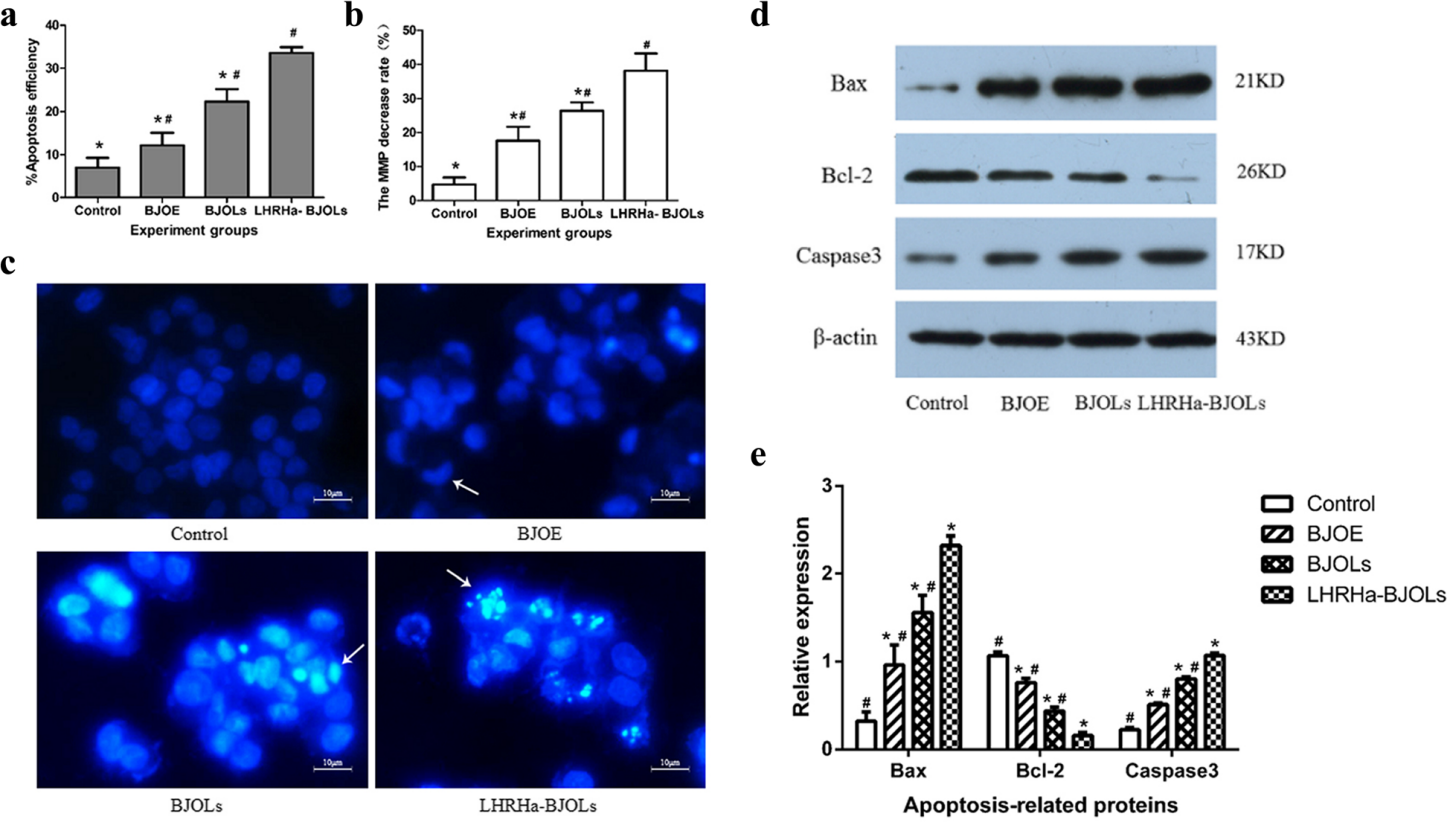
Apoptosis efficiency in A2780/DDP cells in different treatment group. **a** The percentage of apoptosis cells was determined by flow cytometry (FCM) 24 h after treatment. The cell apoptosis rate was higher in all the treatment groups in compared with control group (*p* < 0.05), the highest apoptosis rate was observed in LHRHa-BJOLs group (*p* < 0.05). **b** Mitochondrial membrane potential (MMP) decrease rate detected by FCM. The cell MMP was lower in all the treatment groups in compared with control group (*p* < 0.05), LHRHa-BJOLs group had the lowest MMP in compared with other treatment groups (*p* < 0.05). **c** Cell apoptosis detected by Hoechst 33258 fluorescent staining. PBS group exhibited normal nuclear morphology, nuclei fluoresced faint blue and the color was homogenous. BJOE group also exhibited faint blue fluorescence emission, only a few chromatin condensation were observed. BJOLs group exhibited moderate blue fluorescence emission and some nuclear condensation and morphological changes such as chromatin condensation and fragmentation. LHRHa-BJOLs group exhibited the highest blue fluorescence emission, chromatin condensation and fragmentation could be visualized in many cells, indicating that LHRHa modification of the BJOLs yields the highest apoptosis efficiency. Compared with LHRHa-BJOLs group *p < 0.05; compared with control group, #*p* < 0.05. **d** Western blot analysis of the expression bcl-2, bax and caspase 3 protein in A2780/DDP cells after different treatments, LHRHa-BJOLs group showed more prominent bands of bax and caspase 3 and less band of bcl-2 than other treatment groups. **e** The expression levels of bcl-2, bax and caspase 3 protein in different treatments groups. LHRHa-BJOLs group manifested the highest expression of bax and caspase-3 and the lowest expression of bcl-2, and the differences were statistically significant. Compared with control group, *p < 0.05; compared with LHRHa-BJOLs group, #*p* < 0.05

FCM analysis was used to evaluate the percentages of apoptotic cells following each treatment (Fig. 4a). The results demonstrated that BJOE, BJOLs and LHRHa-BJOLs induced significantly more apoptosis of A2780/DDP cells than PBS. The highest apoptosis rate was observed in LHRHa-BJOLs group, with (33.36 ± 1.31) % after 24 h incubation, and was significantly higher than in BJOLs (22.34 ± 2.88) %, BJOE (12.15 ± 2.93) % and control groups (6.95 ± 2.29) % (*p* < 0.05), indicating the significant increase of the cell apoptosis efficiency by LHRHa-BJOLs treatment. Quantitative analysis of the mitochondrial membrane potential (MMP) decrease by FCM analysis for these treatment groups also yielded consistent conclusions. According to Fig. 4b, the MMP decrease rate in PBS, BJOE, BJOLs and LHRHa-BJOLs groups were (4.70 ± 2.11 %) %, (17.59 ± 4.15) %, (25.69 ± 2.95) % and (38.16 ± 5.08) %, respectively. Compared with other treatment groups, LHRHa-BJOLs group exhibited the highest MMP decrease rate (*p* < 0.05).

Cell apoptosis was also detected by Hoechst 33258 fluorescent staining. Figure 4c shows the fluorescence microscopic images acquired for the four groups. PBS group exhibited normal nuclear morphology, nuclei fluoresced faint blue and the color was homogenous. BJOE group also exhibited faint blue fluorescence emission, only a few chromatin condensation were observed, indicating that application of BJOE can induce A2780/DDP cell apoptosis slightly. BJOLs group exhibited moderate blue fluorescence emission and some nuclear condensation and morphological changes such as chromatin condensation and fragmentation were observed, indicating that BJOLs may enhance cell apoptosis even without the use of a targeting vector. LHRHa-BJOLs group exhibited the highest blue fluorescence emission and chromatin condensation and fragmentation could be visualized in many cells, indicating that LHRHa modification of the BJOLs yields the highest apoptosis efficiency.

Tumor apoptosis related protein bcl-2, bax and caspase 3 expression after different treatment was evaluated by western blot assay. As shown in Fig. 4d, cells treated with LHRHa-BJOLs showed more prominent bands of bax and caspase 3 and less band of bcl-2 than other treatment groups. Figure 4e shows the expression levels of bcl-2, bax and caspase-3 respectively for four treatment groups. The indexes of bcl-2/β-action for PBS, BJOE, BJOLs and LHRHa-BJOLs groups were (1.07 ± 0.04), (0.76 ± 0.05), (0.43 ± 0.05), and (0.16 ± 0.03), respectively. Whereas the indexes of bax/β-action were (0.32 ± 0.11), (0.96 ± 0.23), (1.56 ± 0.19) and (2.32 ± 0.11), and the indexes of caspase 3/β-action were (0.22 ± 0.02), (0.51 ± 0.02), (0.80 ± 0.03) and (1.07 ± 0.03), respectively. Compared with control group, the expression levels of bax and caspase 3 were upregulated and bcl-2 was downregulated in BJOE, BJOLs and LHRHa-BJOLs groups. Compared with other treatment groups, LHRHa-BJOLs group exhibited the highest expression of bax and caspase-3 and the lowest expression of bcl-2, and the differences were statistically significant (*p* < 0.05).

### Establishment of ovarian cancer-bearing mice

The ovarian cancer-bearing mice were successfully established by subcutaneously injection of A2780/DDP cells in nude mice. The mice were in good condition in the early days after inoculation, but the mobility and eating habits began to decline from about 3 weeks after inoculation, especially in control and BJOE groups. During the period of treatment, one mouse in BJOE group exhibited diarrhea and reduced activity at 14 days after tumor inoculation (one day before the first treatment) and recuperated within the next 2 days; while one mouse in BJOLs group showed diarrhea and decreased appetite at 22 days after tumor inoculation (1 day after the third treatment) and returned to the average level in the next day. By the end of the experiment, all the animals died of the progressive tumor growth with no obvious evidence of toxicity.

### Measurement of tumor volume after in vivo treatment

**Fig. 5:**
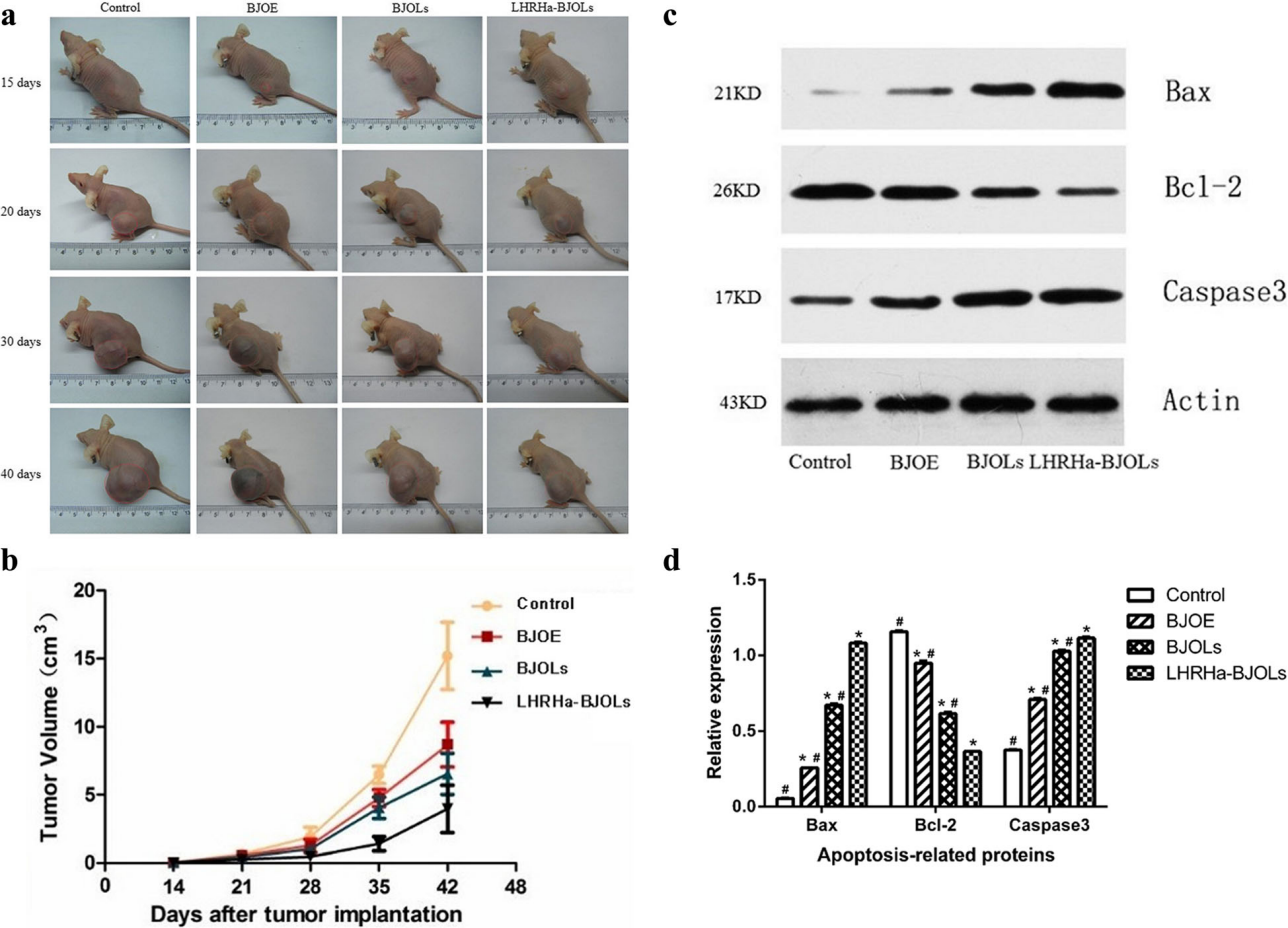
Growth inhibition of A2780/DDP derived tumors in the tumor xenograft models. **a** Typical photographs of mice bearing A2780/DDP tumors after the different treatment. Representative photographs from each group. **b** The tumor growth curve for the different treatment groups (*n* = 5 in each group). The tumors’ volume in the experiment groups were significantly smaller compared to that in the PBS control group (*p* < 0.05), and the mice in the treatment group of LHRHa-BJOLs manifested the slowest tumor growth rate. **c** Western blot analysis of the expression bcl-2, bax and caspase 3 protein in tumor tissue after different treatments, LHRHa-BJOLs group showed more prominent bands of bax and caspase 3 and less band of bcl-2 than other treatment groups (*n* = 3 in each group). **d** The expression levels of bcl-2, bax and caspase 3 protein in different treatments groups. LHRHa-BJOLs group has the highest expression of bax and caspase-3 and the lowest expression of bcl-2, and the differences were statistically significant (*n* = 3 in each group). Compared with control group, *p < 0.05; Compared with LHRHa-BJOLs group, #*p* < 0.05

The tumor nodules were palpable 7 days and visible 14 days after inoculation, photographic images were also taken after each treatment session (Fig. 5a), the LHRHa-BJOLs group exhibited the lowest level of tumor volume, and this was further verified by calculating the relative tumor volumes of different treatment groups at different time. Figure 5b plots the tumor growth curve for the different treatment groups, according to the figure, the tumors’ volume in the experiment groups were significantly smaller compared to that in the PBS control group (p < 0.05), and the mice in the treatment group of LHRHa-BJOLs manifested the slowest tumor growth rate.

### Expression of Bcl-2, Bax and caspase 3 in tumor tissue

Tumor apoptosis related proteins, bcl-2, bax and caspase 3, were evaluated by western blot analysis. Figure 5c and d shows the expression levels of bcl-2, bax and caspase 3 after different treatment groups. The indexes of bcl-2/β-action for PBS, BJOE, BJOLs and LHRHa-BJOLs groups were (1.16 ± 0.01), (0.95 ± 0.02), (0.62 ± 0.01) and (0.37 ± 0.001), respectively. Whereas the indexes of bax/β-action were (0.06 ± 0.001), (0.26 ± 0.001), (0.67 ± 0.01) and (1.08 ± 0.01), and the indexes of caspase 3/β-action were (0.38 ± 0.001), (0.71 ± 0.01), (1.03 ± 0.01) and (1.12 ± 0.01), respectively. Compared with the other treatment groups, LHRHa-BJOLs group has the highest expression of bax and caspase-3 and the lowest expression of bcl-2, and the differences were statistically significant (*p* < 0.05). These results indicate that LHRHa-BJOLs significantly upregulated pro-apoptotic protein bax and downregulated anti-apoptotic protein bcl-2 expression, and increase apoptosis related protein caspase 3 expression, which were consistent with the in vitro results.

### Survival analysis after in vivo treatment

**Fig. 6:**
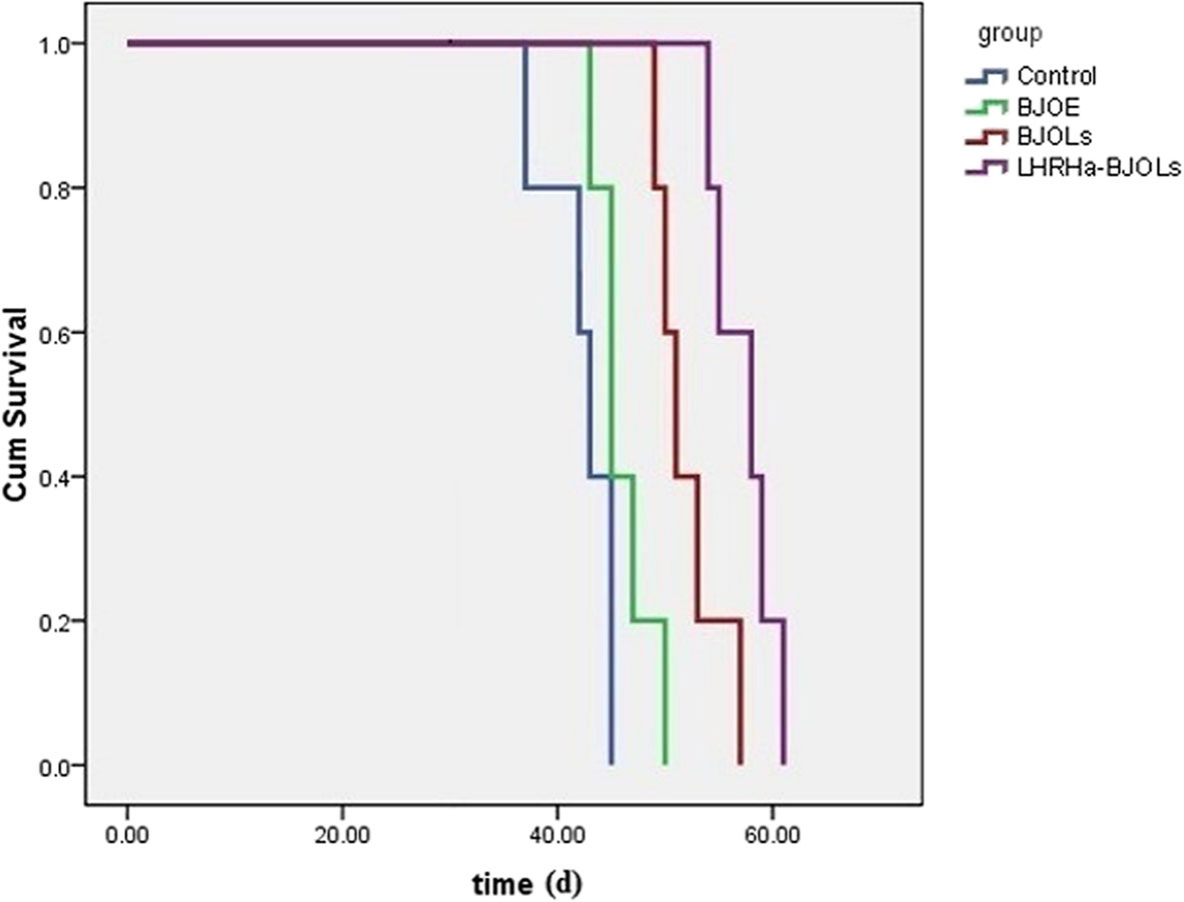
The survival curve of the mice in different treatment groups (*n* = 5 in each group). The LHRHa-BJOLs group exhibited the longest survival time which was significantly longer than other three groups (*p* < 0.05)

By the end of the experiment, all the mice died of the progressive tumor growth. Figure 6 plots the survival curves, the control group had a median survival time of 42.40 ± 1.47 days, while the other three treatment groups exhibited different levels of the extended survival time, 46.00 ± 1.18 days in BJOE group, 52.00 ± 1.40 days in BJOLs group and 58.60 ± 1.03 days in LHRHa-BJOLs group. The BJOLs and LHRHa-BJOLs groups showed a superior therapeutic outcome with the median survival time significantly longer than other two groups, and the LHRHa-BJOLs group exhibited the longest survival time which was also significantly longer than BJOLs group. Additionally, compared with control group, BJOE also yielded the increased survival time to some extent, but the difference was not significant, implying that direct application of BJOE intravenously without liposomes mediation cannot prolong the survival time significantly, although it can induce significantly more tumor cells apoptosis related protein expression as described above.

## Discussion

Brucea javanica oil (BJO) is a complex mixture of fatty acids and its derivatives. It’s main component are oleic acid and linoleic acid [9], and has been used to treat various tumors for many years in China [26–29]. However, its non-specific distribution and low therapeutic index are two main reasons for poor prognosis in tumor therapy. Liposomes is regarded as a promising drug carrier with good biocompatibility, biodegradability and low cytotoxicity for cancer therapeutics [30], and it is also beneficial for the parenteral delivery of insoluble drugs because it’s better stability in plasma [12, 13]. LHRHa peptide has been used to target the corresponding LHRHR over-expressed in the plasma membrane of ovarian cancer [15, 16, 31]. and we also have successfully coated microbubbles (MBs) with LHRHa to enhance the binding affinity with ovarian cancer cells [20–22]. Therefore, we hypothesize that new LHRHa-targeted and BJO-loaded liposomes (LHRHa-BJOLs) will facilitate drug deposition at the tumor site for the enhanced therapeutic outcome. The results obtained in this in vitro and in vivo study are positive and in agreement with our study design, rendering the proposed LHRHa-BJOLs is a promising and novel targeting therapy strategy for the treatment of ovarian cancer.

In the present study, LHRHa-BJOLs have been successfully developed, and the colloidal gold immunoassay test had verified that LHRHa peptide was indeed conjugated on the surface of the liposomes. Particle size, zeta potential and encapsulation efficiency are the properties that influence the biopharmaceutical characteristics and stability of a liposomes. The smaller liposomes can more easily evade the reticuloendothelial system (RES) and prolong the time in circulation [7, 32, 33]. It has been reported that particle size of the drug carrier should be bigger than 10 nm to escape the first-pass elimination by the kidney [34–36], while with particle size less than 200 nm could increase drug accumulation in the tumor via “enhanced permeability and retention (EPR)” effect [25, 37–40]. Meanwhile, the particle size also makes an effect on their interactions with the target cells [40]. Therefore, the LHRHa-BJOLs synthesized in this study had an optimal size (155.1 ± 14.5 nm) for the tumor targeting by the EPR effect. The zeta-potential is a widely accepted parameter to represent the surface charge of the liposomes, and could influence on both its colloidal stability in suspension and its interaction with cells [41]. For LHRHa-BJOLs synthesized in this study, the zeta potentials was - (24.1 ± 0.54) mV, demonstrating that the negatively charged DSPE-PEG_2000_ was successfully inserted into the outer monolayer of the vesicles [40]. Furthermore, the zeta potential was within the range from −20 mV to −30 mV, considering to be stable in aqueous environment without flocculation [42]. Additionally, the encapsulation efficiency was (93.6 ± 1.04) % before conjugation, and declined to (92.2 ± 1.59) % after conjugation, similar to that reported by Cui et al. [10]. The slight drug loss might be due to the release of BJO from the liposomes in the conjugation process, during which repeated and prolonged stirring was applied [41].

To demonstrate the specific cell binding and internalization of LHRHa-Lip, LHRHR positive A2780/DDP cells were chosen as target cells, while LHRHR negative SKOV3 cells were applied as negative control, and the non-targeted liposomes was also prepared and used as the control. LSCM analysis revealed that the fluorescence intensity in A2780/DDP cells plus LHRHa-C6Ls group was stronger than that in A2780/DDP cells plus C6Ls and SKOV3 cells plus LHRHa-C6Ls groups, this is associated with the LHRHR targeting ability of LHRHa, and indicated that LHRHa conjugation does promote the entry of the liposomes into the LHRHR overexpressed cells by receptor-mediated endocytosis [43]. FCM data further demonstrated that LHRHa-C6Ls resulted in significantly higher cellular uptake by A2780/DDP cells. All these results further suggested that LHRHa-Lip could enhance the specific cell binding and cellular uptake in A2780/DDP cells due to the mediating of LHRHa, and depending on the LHRHR expression level on cell surface as well.

BJOE, BJOLs and LHRHa-BJOLs were found to inhibit A2780/DDP cells proliferation and induce cell apoptosis in some extent, and the LHRHa-BJOLs exhibited the strongest inhibitory and apoptosis effect, this was consistent with the results of cellular uptake discussed above. Furthermore, the in vivo study showed that the median survival time of the animal in LHRHa-BJOLs group was significantly longer than that in BJOLs and BJOE groups at comparable dose of BJO. This may be a consequence of the targeted deposit of LHRHa-BJOLs in the tumor tissue. The mechanisms of this superior ovarian cancer targeting ability of LHRHa-Lip has not been fully understood yet. We believe that this mechanisms might be associated with at least the following contributing factors. First of all, LHRHa acted as an anchor to actively hold the liposomes specifically to the tumor site and promote the receptor-mediated endocytosis. Second, the DSPE-PEG2000 anchored to the surface of LHRHa-Lip are able to avoid rapid uptake by the RES, thus prolonging the circulation time and resulting in a higher accumulation of the liposomes in the tumor vasculature [44]. Third, the optimal particle size of the LHRHa-Lip plays an important role in the increased accumulation by the EPR effect as discussed above. Forth, LHRHa-Lip improve the pharmacokinetic profile of BJO due to the PEGylated materials [45, 46], which ultimately results in a higher accumulation in tumors. The results might further indicated that both active and passive tumor targeting mechanisms were involved in LHRHa-Lip accumulation within tumor, in which active targeting was achieved by LHRHa conjugation to liposomes and passive targeting was attributed to the EPR effect of liposomes [47].

To further investigate the mechanisms underlying the enhanced tumor-inhibition activity of LHRHa-BJOLs, tumor apoptosis was also evaluated by western blot analysis. Activation of bax and caspase 3, inhibition of bcl-2 expression, and decline of MMP are crucial findings, the LHRHa-BJOLs group exhibited significantly more changes than the other groups both in vitro and in vivo. We speculated that the following possible mechanisms might be involved in the enhanced antitumor efficacy of LHRHa-BJOLs. Firstly, LHRHa modification and smaller particle size enhanced the accumulation of BJO in tumor cells which was discussed above, and this is the prerequisite for the BJO to function. Secondly, LHRHa-BJOLs upregulated bax, an important pro-apoptotic protein in mitochondrial pathway [48], and downregulated bcl-2, one member of the anti-apoptotic bcl-2 family proteins and critical determinants of mitochondrial-dependent caspase activation [49]. As a result, the mitochondrial-initiated apoptosis was promoted, and this was also confirmed by the reduced MMP of A2780/DDP cells, which is the result of a mitochondrial leak by opening the permeability transition pores [44]. Thirdly, LHRHa-BJOLs upregulated caspase 3, an important promoters of the death receptor-mediated apoptosis [50, 51], and resulted in activation of the death receptor pathway. Taken together, it could be proposed that LHRHa-BJOLs induced apoptosis in A2780/DDP cells in vitro or in ovarian tumor tissue in vivo via simultaneous activation of the death receptor pathway and the mitochondrial pathway.

Therefore, the LHRHa-BJOLs exhibited a promising ovarian cancer cells targeting capability and enhanced therapeutic efficiency both in vitro and in vivo experiments. However, this study also has some limitations. Firstly, BJO is a complex mixture of fatty acids and its derivatives, and its main activity components are oleic acid and linoleic acid with content of 63.3 and 21.2 % [10], whether the anti-tumor effect of BJO in ovarian cancer was result from the existence of oleic acid or linoleic acid still needed to be further verified. Secondly, we fabricated the targeted liposomes via avidin–biotin interactions, although this method has been demonstrated successfully in animal models, its clinical feasibility is challenged by the unwanted immunogenicity. To overcome this limitation, one possible technical approach is to design avidin in the less immunogenic forms, and the other approach is to use other covalent and non-covalent binding strategies to synthesize tumor targeting liposomes. Further research and validation efforts are required before these technical advances can be implemented in a clinical setting. Finally, no complete recovery was observed for any treated mice in this study, because we chose to initiate the treatment on the 15^th^ day after tumor cell inoculation when the tumors were readily palpable and stop the treatment on the 27^th^ day after inoculation. In order to further improve the outcome, we will consider the optimal therapeutic strategies in the future, such as earlier initiation of the treatment, increase of dose and prolongation of treatment.

## Conclusions

In summary, we have demonstrated successful synthesis of a new LHRHa modified and BJO-loaded liposomes (LHRHa-BJOLs), which have a uniformly spherical shape, appropriate particle size and zeta potential, as well as a high encapsulation efficiency. Compared to non-targeted liposomes (BJOLs) and free BJOE, the LHRHa-BJOLs can significantly enhance the specific intracellular uptake, cell inhibition and apoptosis in A2780/DDP cells in vitro. Meanwhile, LHRHa-BJOLs have a significantly stronger activity of inhibiting tumor growth, inducing tumor apoptosis and prolonging survival time in ovarian cancer-bearing mice model in vivo. LHRHa-BJOLs can provide a more effective chemotherapy strategy for the LHRHR-positive ovarian cancer.

## Abbreviations

BJO: Brucea javanica oil
BJOLs: Brucea javanica oil liposomes
C6Ls: Coumarin-6 loaded liposomes
EE: Encapsulation efficiency
EPR: Enhanced permeability and retention
FCM: Flow cytometry
LHRHa: Luteinizing hormone releasing hormone analogue
LHRHR: Luteinizing hormone releasing hormone receptor
LSCM: Laser scanning confocal microscope
MFI: Mean fluorescent intensity
MMP: Mitochondrial membrane potential
OD: Optical density
PBS: Phosphate buffered saline
TEM: Transmission electron microscopy
